# Microsurgical technique for tracheostomy in the rat

**DOI:** 10.1016/j.mex.2017.10.010

**Published:** 2017-11-16

**Authors:** Michael George Zaki Ghali

**Affiliations:** Department of Neurobiology & Anatomy, Drexel University College of Medicine, 2900 Queen Lane, Philadelphia, PA, 19129, USA

**Keywords:** Tracheostomy, Tracheotomy, Airway access, Ventilation, Technique, Rat

## Abstract

Tracheostomy is used to obtain airway access to allow for mechanical ventilation in experimental animal models. We present a microsurgical atlas of our technique for tracheostomy in the adult rat.

## Graphical abstract

**Figure:**
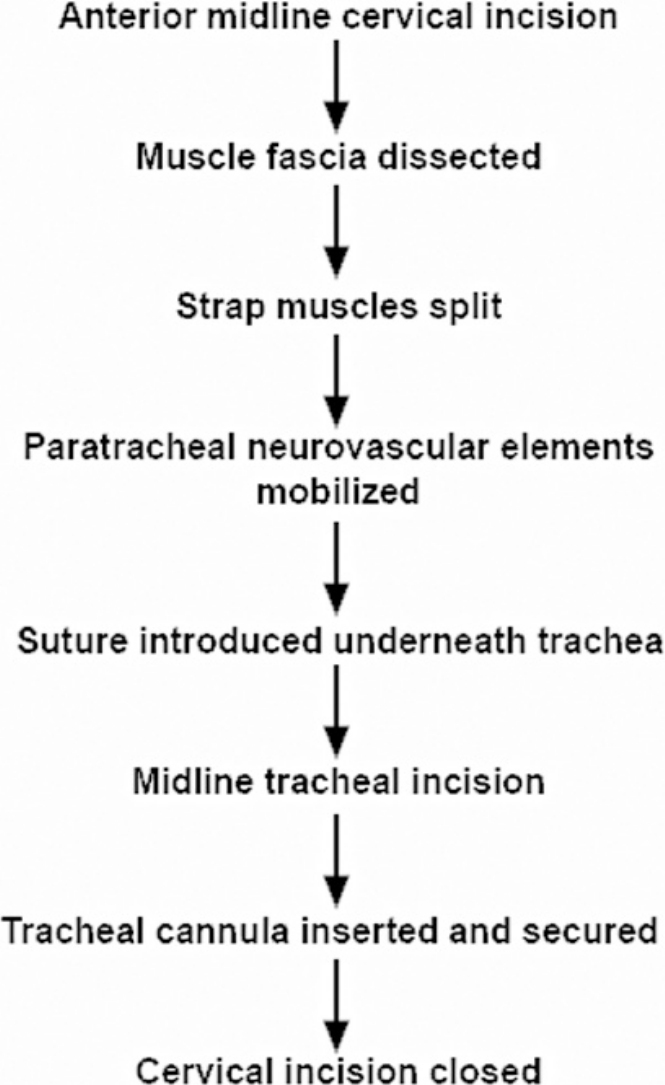

## Introduction

Tracheostomy achieves safe airway access, provides the investigator with ventilatory control during *in vivo* acute or terminal physiological experiments, and serves as an alternative to endotracheal intubation. Our technique is presented in descriptive and illustrative detail, as we have employed in our previous studies [[1], [2], [3], [4], [5], [6]].

## Methods

All procedures were approved by the Drexel University Institutional Animal Care and Use Committee, which oversees Drexel University’s AAALAC International-accredited animal program. Ten spontaneously-breathing, Sprague-Dawley adult male rats (340–380 g) were anesthetized with isoflurane (Matrix; 4–5% induction, 1.85–2.15% maintenance) vaporized in O_2_ via a snout mask. The electrocardiogram (EKG) was measured via three small subcutaneous electrodes using conventional amplification and filtering (Neurolog; Digitimer, Hertfordshire, UK) and monitored using an audio amplifier (model AM10; Grass Instruments) and oscilloscope. Anesthetic depth was maintained at a level such that withdrawal reflexes and changes in heart rate in response to pinches of the distal hind limbs were absent.

**Fig. 1:**
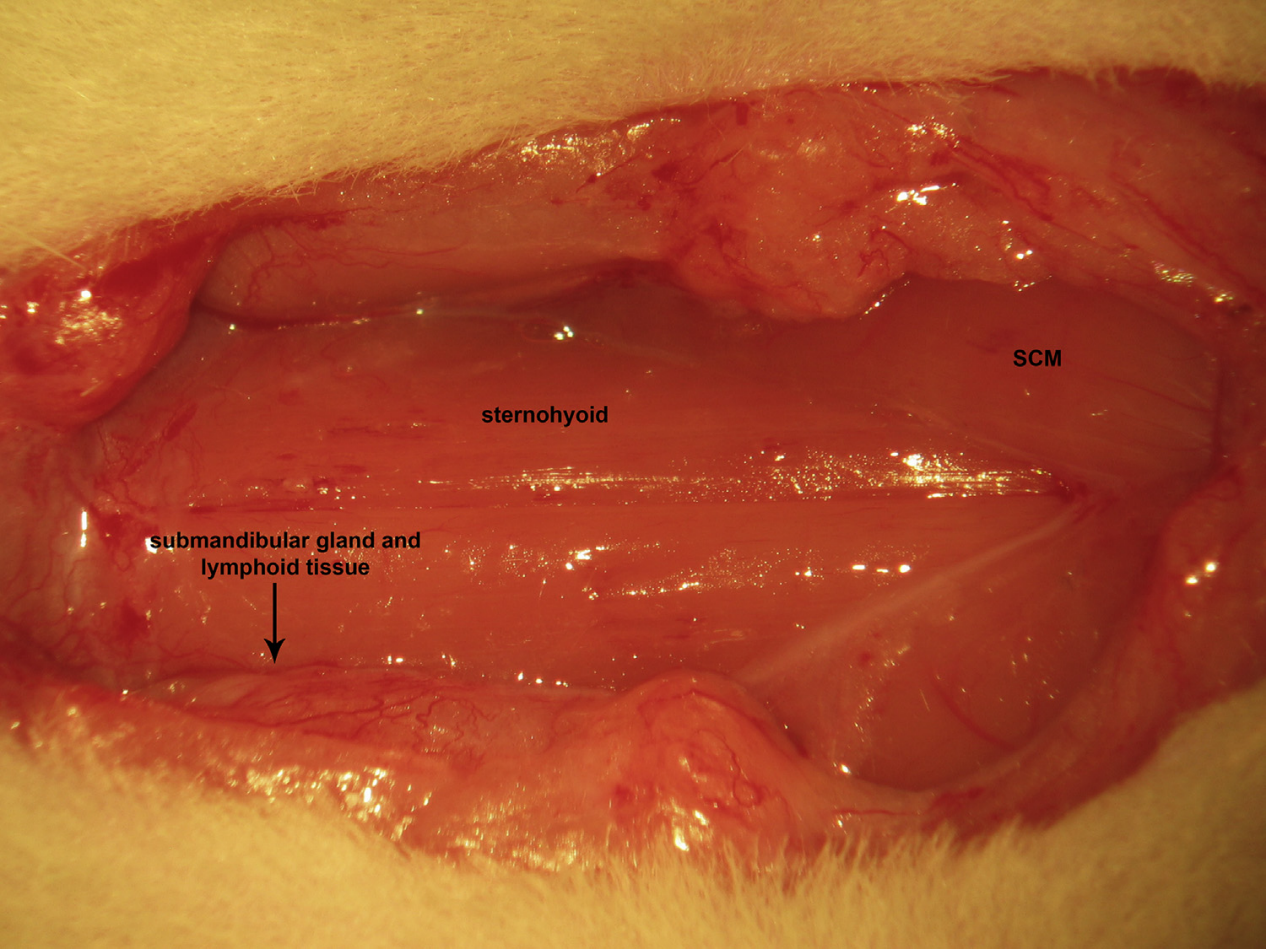
A ventral midline cervical incision is made from the manubrium sterni to the level of the hyoid bone. Blunt dissection of cervical soft tissue exposes the sternocleidomastoid and sternohyoid muscles.

**Fig. 2:**
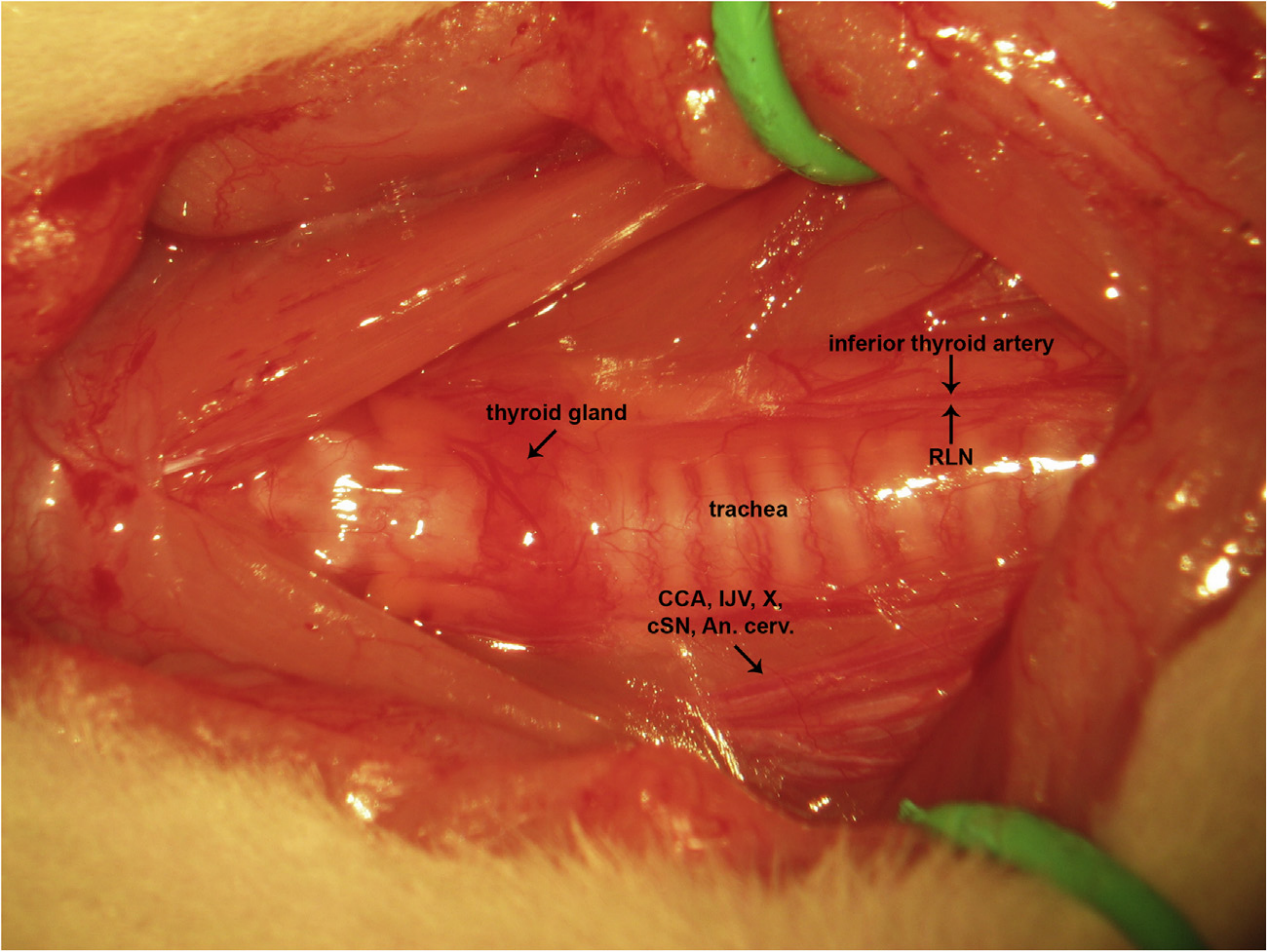
Fascia overlying the paired sternohyoids is split in the midline and each muscle of the pair is retracted laterally to expose the trachea.

**Fig. 3:**
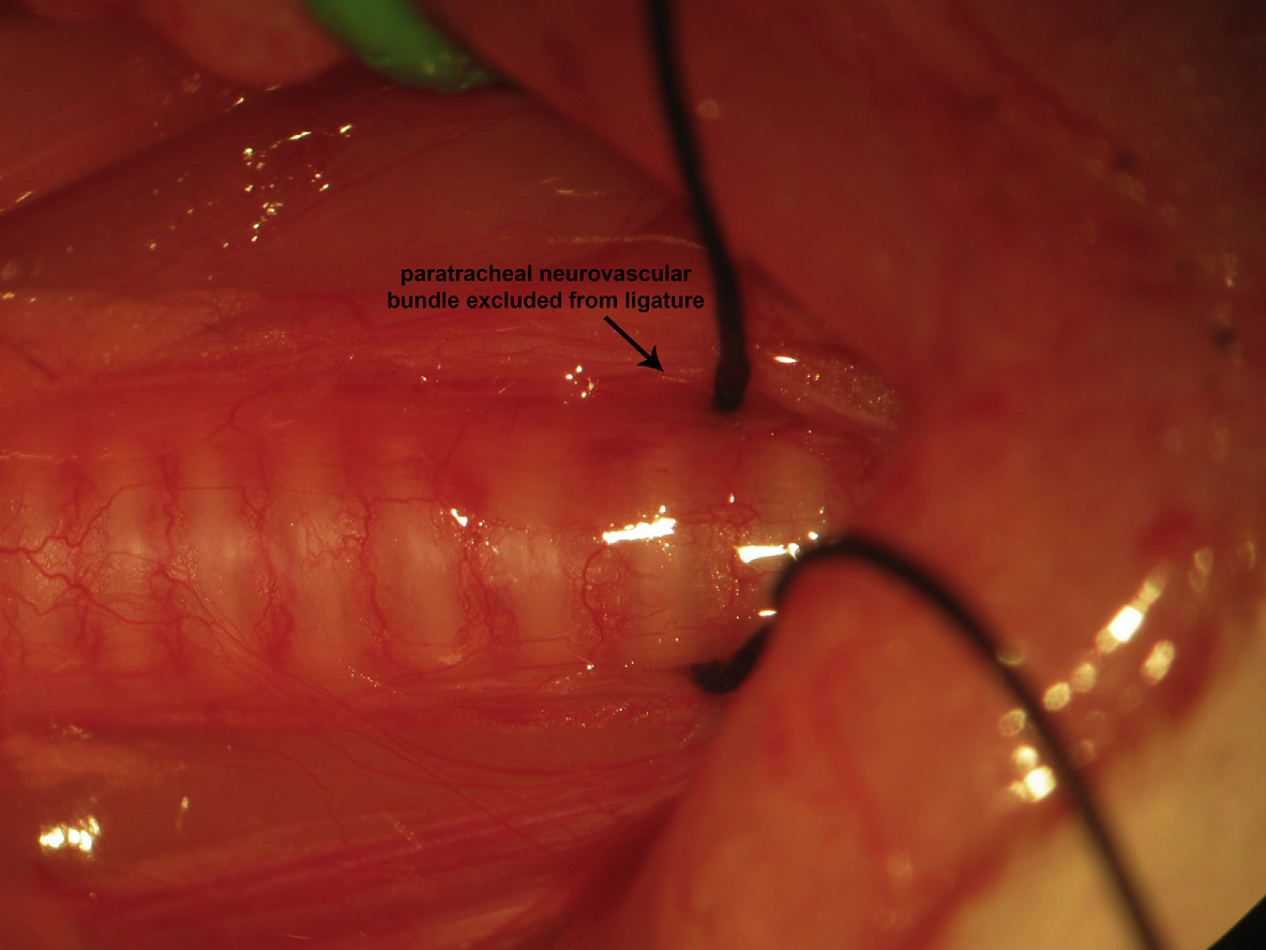
The inferior thyroid artery and recurrent laryngeal nerve are mobilized from the trachea and a suture is placed underneath the trachea exclusive of this paratracheal neurovascular bundle.

**Fig. 4:**
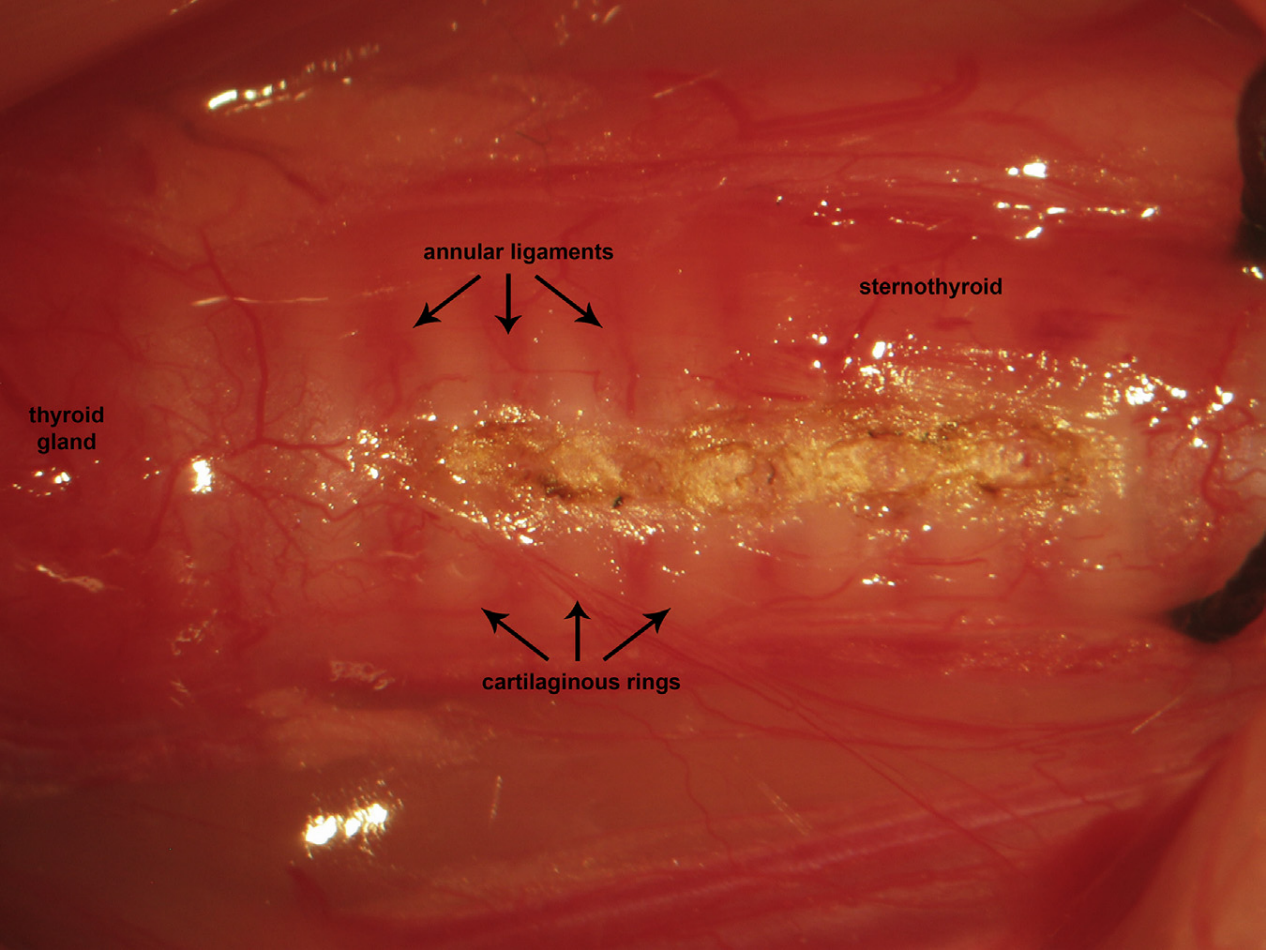
Electrocautery is used to perform a midline incision of the trachea sufficiently caudal to the thyroid gland (∼2 cartilaginous tracheal rings) so as to prevent inadvertent thermal damage.

**Fig. 5:**
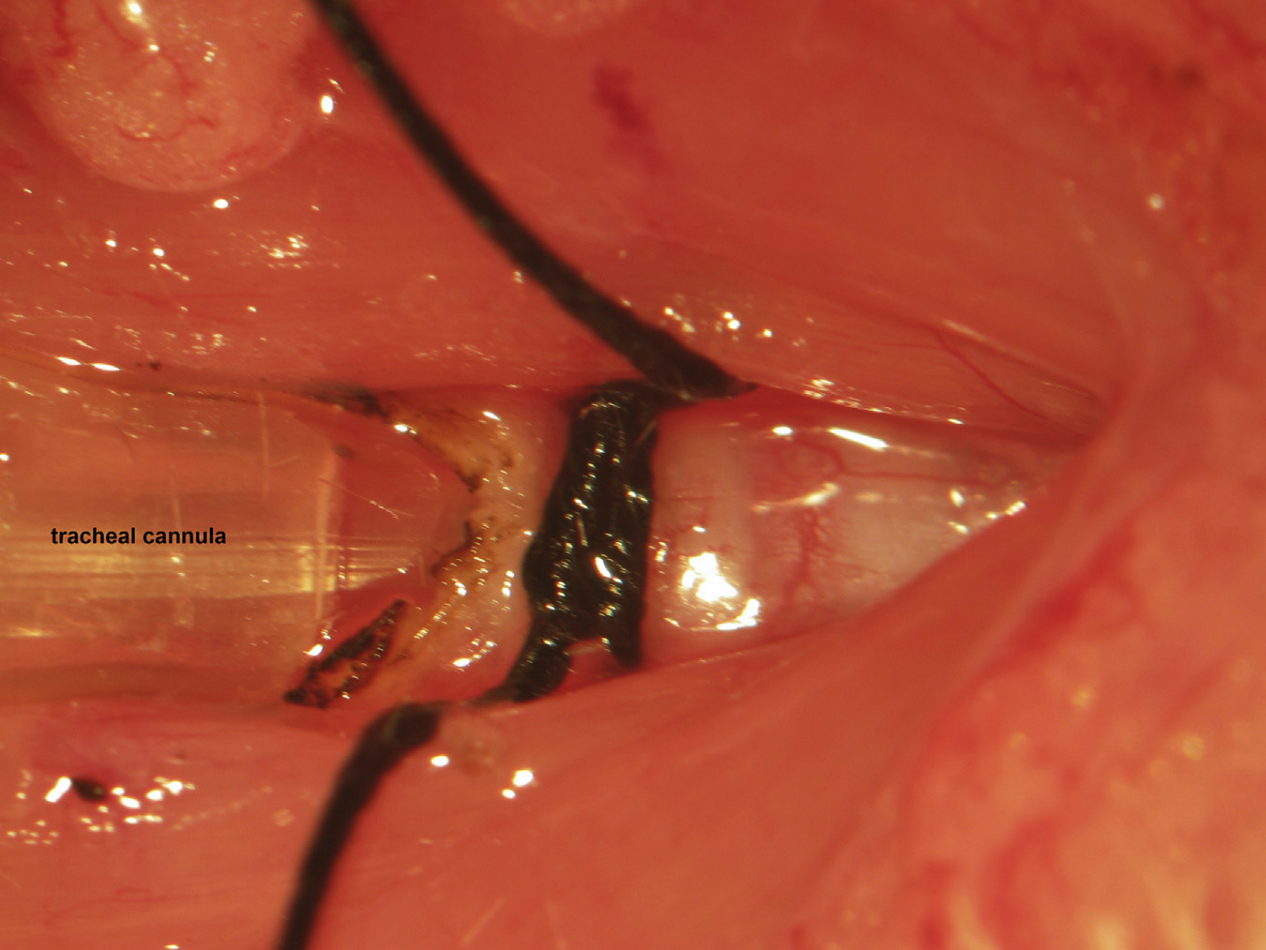
A cannula is inserted into the trachea and secured with 3-0 silk suture.

**Fig. 6:**
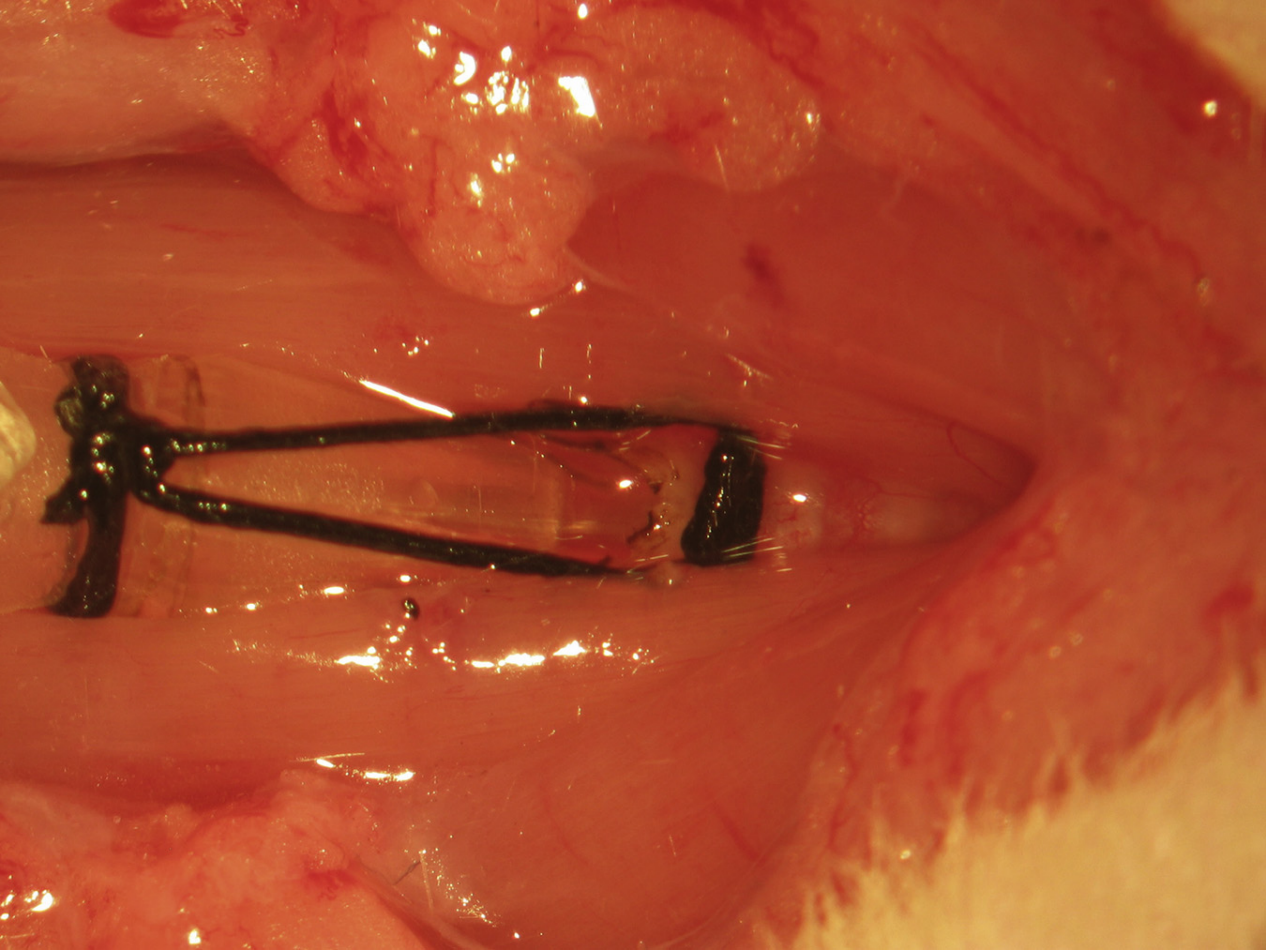
A “knot anchor” is created and used to secure the cannula to the trachea.

**Fig. 7:**
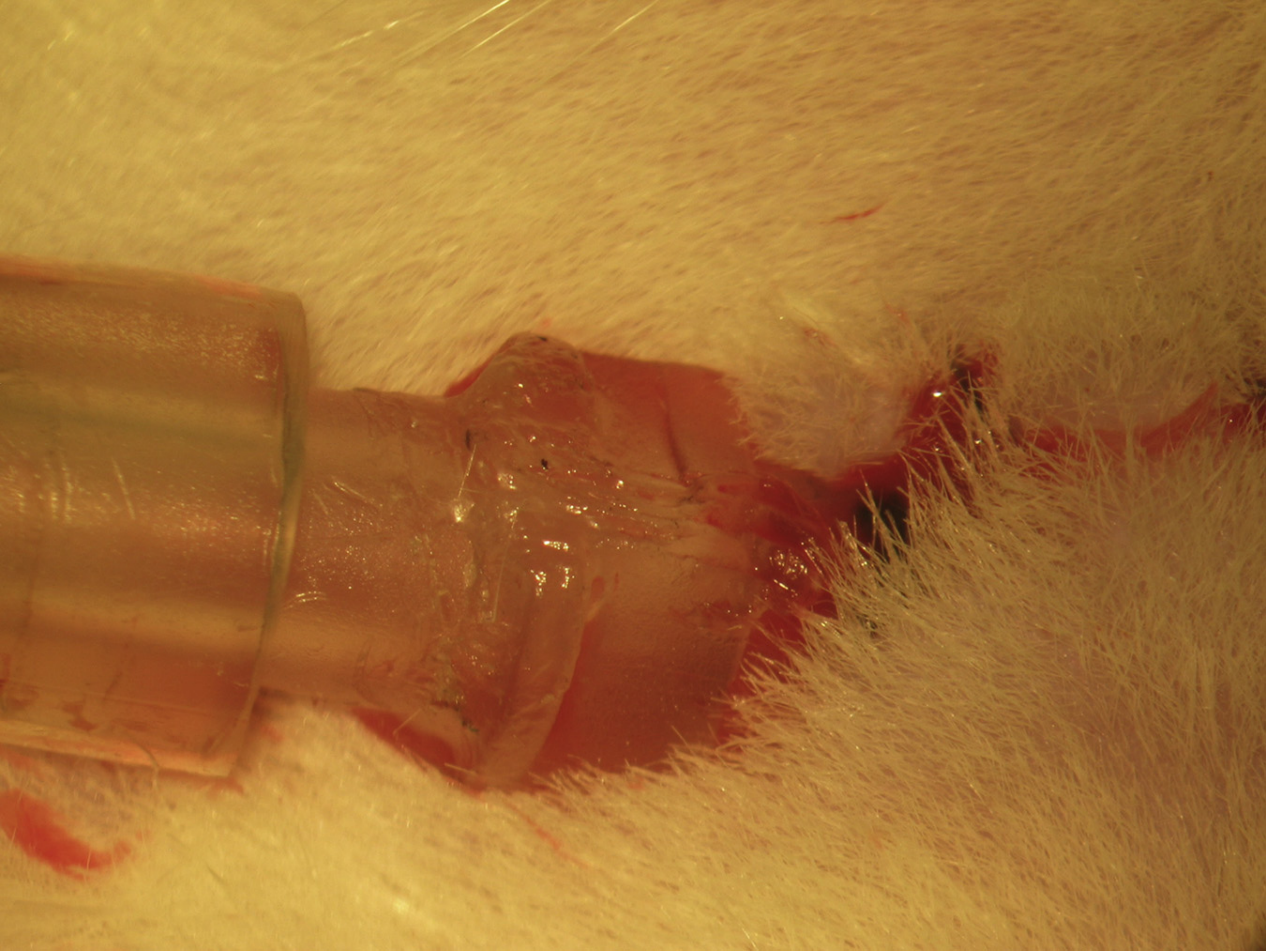
The cervical incision is sutured tightly using continuous 4-0 silk suture.

**Fig. 8:**
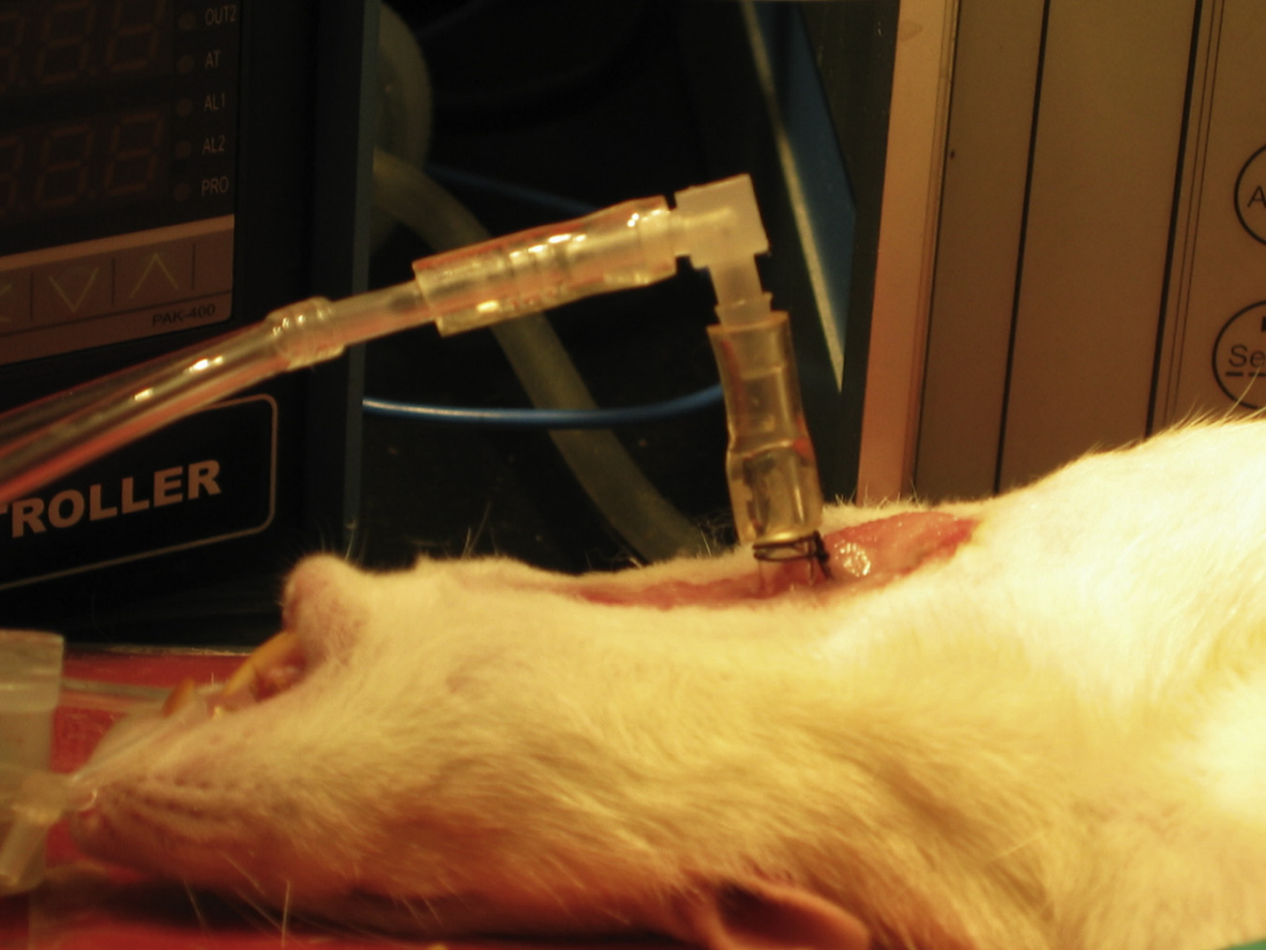
Ventrally-curved tracheal cannula.

A ventral midline cervical incision is made from the manubrium sterni to the level of the hyoid bone. Blunt dissection of cervical soft tissue exposes the sternocleidomastoid and sternohyoid muscles (Fig. 1). Fascia overlying the paired sternohyoids is split in the midline and each muscle of the pair is retracted laterally to expose the trachea (Fig. 2). The inferior thyroid artery and recurrent laryngeal nerve are mobilized from the trachea and a suture is placed underneath the trachea exclusive of this paratracheal neurovascular bundle (Fig. 3). Electrocautery is used to perform a midline incision of the trachea sufficiently caudal to the thyroid gland (∼2 cartilaginous tracheal rings) so as to prevent inadvertent thermal damage (Fig. 4). A tracheal cannula is inserted into the trachea and secured with 3-0 silk suture (Fig. 5). A “knot anchor” is created and used to secure the cannula to the trachea (Fig. 6). The cervical incision is sutured tightly using continuous 4-0 silk suture (Fig. 7). A ventrally-curved tracheal cannula is used in the case cervical vertebral corpectomy is planned (Fig. 8).

## Conclusion

We present our technique for tracheostomy to achieve airway access and permit mechanical ventilation, which we employ in acute physiological experiments investigating respiratory-related neural activity. It is important to perform tracheostomy without neurovascular injury and without including the recurrent laryngeal nerves in the ligature securing the tracheal cannula in order to avoid triggering of vagal reflexes which may affect cardiovascular and respiratory neural network dynamics.

## Conflicts of interest

None to disclose.

## Funding

Drexel University College of Medicine
